# MicroRNAs, New Players in the Plant–Nematode Interaction

**DOI:** 10.3389/fpls.2019.01180

**Published:** 2019-10-17

**Authors:** Stéphanie Jaubert-Possamai, Yara Noureddine, Bruno Favery

**Affiliations:** ISA, INRA, Université Côte d’Azur, CNRS, Sophia Antipolis, France

**Keywords:** root-knot nematodes, cyst nematodes, galls, syncytium, microRNAs, siRNAs

## Abstract

Plant-parasitic root-knot and cyst nematodes are microscopic worms that cause severe damage to crops and induce major agricultural losses worldwide. These parasites penetrate into host roots and induce the formation of specialized feeding structures, which supply the resources required for nematode development. Root-knot nematodes induce the redifferentiation of five to seven root cells into giant multinucleate feeding cells, whereas cyst nematodes induce the formation of a multinucleate syncytium by targeting a single root cell. Transcriptomic analyses have shown that the induction of these feeding cells by nematodes involves an extensive reprogramming of gene expression within the targeted root cells. MicroRNAs are small noncoding RNAs that act as key regulators of gene expression in eukaryotes by inducing the posttranscriptional silencing of protein coding genes, including many genes encoding transcription factors. A number of microRNAs (miRNAs) displaying changes in expression in root cells in response to nematode infection have recently been identified in various plant species. Modules consisting of miRNAs and the transcription factors they target were recently shown to be required for correct feeding site formation. Examples include miR396 and *GRF* in soybean syncytia and miR159 and MYB33 in *Arabidopsis* giant cells. Moreover, some conserved miRNA/target modules seem to have similar functions in feeding site formation in different plant species. These miRNAs may be master regulators of the reprogramming of expression occurring during feeding site formation. This review summarizes current knowledge about the role of these plant miRNAs in plant–nematode interactions.

## Introduction

**Figure 1:**
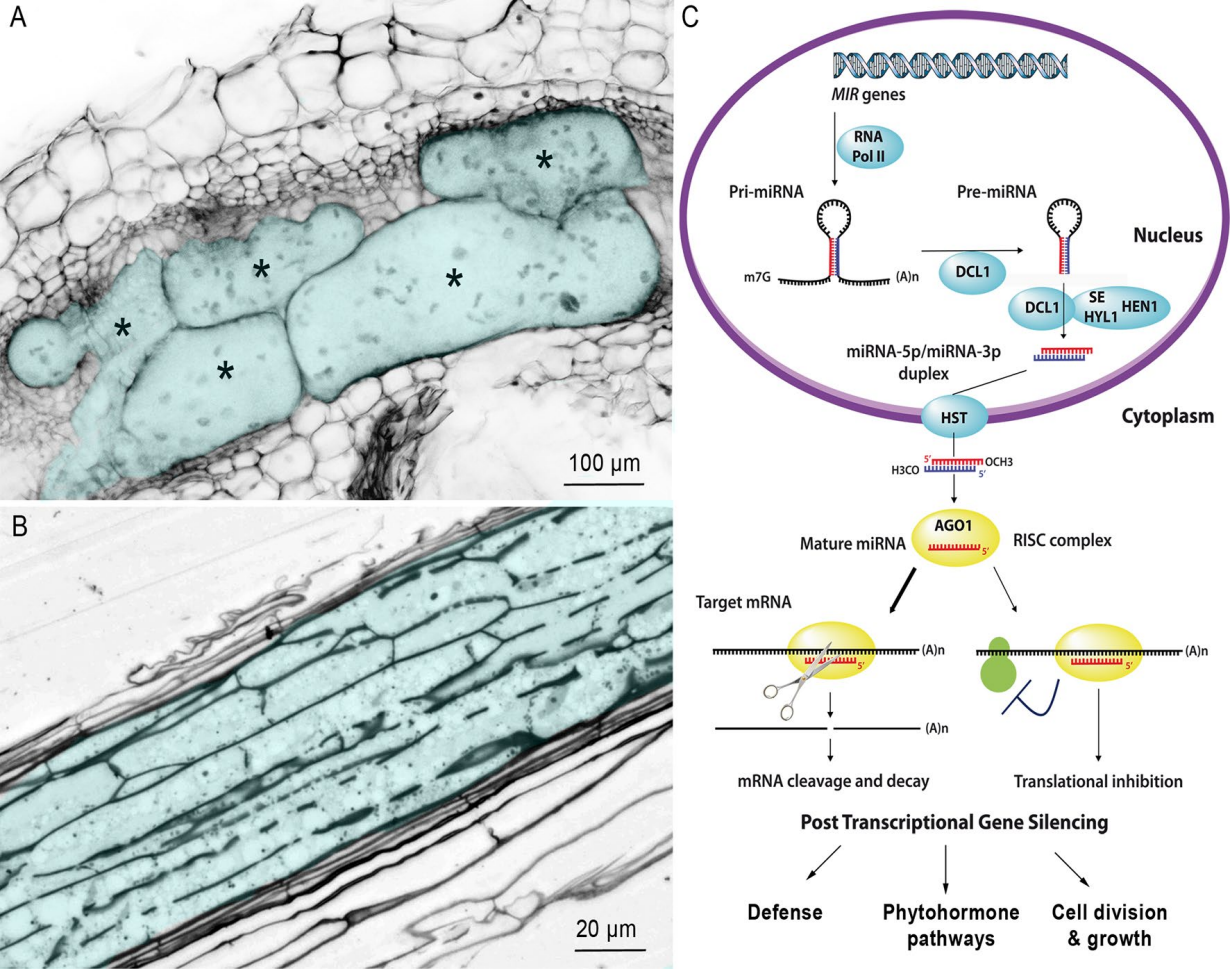
Multinucleate and hypertrophied feeding cells induced by RKN and CN. **(A)** Confocal section of a gall induced by *M. incognita* in *Nicotiana benthamiana*. Galls were fixed and cleared with the BABB method described by Cabrera et al. (2018). Giant cells are colored in blue and marked with an asterisk to differentiate them from surrounding cells of normal size. Bar = 100 µm. **(B)** Longitudinal section of a syncytium induced by the CN *H. schachtii* in Arabidopsis roots, 10 days after inoculation. The syncytium is colored in blue. Bar = 20 µm. **(C)** Simplified biogenesis and mechanism of action of miRNAs in plants. The *MIR* genes are transcribed by RNA polymerase II (RNA Pol II) to generate single-stranded hairpin-containing primary transcripts (pri-miRNA). The pri-miRNA is then cleaved, in the nucleus, by Dicer-like 1 (DCL1), in association with hyponastic leaves 1 (HYL1) and serrate (SE), to produce a precursor miRNA (pre-miRNA). The pre-miRNA is, in turn, cleaved by DCL1 and its cofactors, thus generating a duplex composed of the mature miRNA and its complementary strand. The HUA ENHANCER 1 protein (HEN1) then adds a methyl group to the OH end of each strand of the miRNA duplex, to protect against degradation. The miRNA duplex is then actively transported from the nucleus to the cytosol through interaction with the hasty (HST) exportin. One of the two strands of the duplex is then loaded onto the argonaute 1 (AGO1) protein, the main constituent of the multiprotein RNA-induced silencing complex (RISC). The AGO1-associated strand guides the RISC to target mRNAs by sequence complementarity, resulting in target cleavage or the inhibition of protein synthesis (reviewed by Yu et al., 2017).

Sedentary endoparasitic nematodes are the most damaging plant-parasitic nematodes (PPNs) that cause massive crop yield losses worldwide (Blok et al., 2008). There are two main groups of PPNs: the root-knot nematodes (RKNs) of the genus *Meloidogyne* and the cyst nematodes (CNs) of the genera *Heterodera* and *Globodera* (Jones et al., 2013). After penetrating the root and migrating to the vascular cylinder, mobile second-stage juvenile (J2) selects one (CNs) or a few (RKNs) initial root cells, into which it injects a cocktail of secretions that transform these cells into hypertrophied multinucleate feeding cells that supply nutrients required for nematode development: the giant cells induced by RKNs (**Figure 1A**) or the syncytium induced by CNs (**Figure 1B**).

### Common and Specific Processes Involved in Feeding Site Formation

Both hypertrophied and multinucleate feeding cells are highly active metabolically and have a dense cytoplasm, with a large number of organelles and invaginated cell wall (**Figure 1A, B**) (Grundler et al., 1998; Sobczak and Golinowski, 2011; Favery et al., 2016). They accumulate sugars and amino acids (Hofmann et al., 2010; Baldacci-Cresp et al., 2012). The nuclei and nucleoli of both giant cells and syncytia are larger than normal root cells, due to endoreduplication (de Almeida Engler and Gheysen, 2013). However, these two feeding structures have very different ontogenies. RKN J2 selects five to seven parenchyma cells and induces their dedifferentiation into giant cells through successive mitosis without cytokinesis (Caillaud et al., 2008b). Expansion of giant cells by isotropic growth (Cabrera et al., 2015) together with hyperplasia of the root cells surrounding the giant cells results in a swelling of the root, known as a gall, the characteristic symptom of RKN infection. By contrast, CN J2 targets a single initial root cell. This cell expands within the vascular tissue by progressive cell wall dissolution and incorporation into the syncytium of adjacent cells *via* cytoplasm fusion (Golinowski et al., 1996; Grundler et al., 1998).

Studies of the feeding site formation have greatly benefited from whole-transcriptome analyses. Such analyses were initially developed in the model host plant *Arabidopsis thaliana* and were then extended to various crop species (Escobar et al., 2011; Favery et al., 2016; Yamaguchi et al., 2017). All these analyses showed that feeding site formation involves an extensive reprogramming of gene expression within the root cells targeted by the nematodes. These analyses suggested that CNs and RKNs establish feeding sites by recruiting and/or manipulating several plant functions, including plant defense and phytohormone pathways (Gheysen and Mitchum, 2019), cell wall modification (Sobczak and Golinowski, 2011), cytoskeleton (Caillaud et al., 2008a), and the cell cycle (de Almeida Engler and Gheysen, 2013). These analyses also revealed the conservation of some nematode-responsive genes within the plant kingdom (Portillo et al., 2013).

### MicroRNAs Are Key Regulators of Gene Expression

Plant miRNAs are 20- to 22-nucleotide-long noncoding RNAs (Bartel, 2004) that regulate gene expression through posttranscriptional gene silencing. Plant miRNA precursors are produced from *MIR* genes and are processed by several proteins, including Dicer-like 1 (DCL1), to generate a mature miRNA duplex. One strand of the duplex is loaded into the RNA-induced silencing complex (RISC), in which its sequence complementarity directs gene silencing (**Figure 1C**) (Yu et al., 2017). Perfect miRNA/mRNA complementarity generally induces cleavage of the mRNA at nucleotide position 10 or 11 (Franco-Zorrilla et al., 2007; Bartel, 2009). However, in some cases, such as the miR172/*APETALA2* module in *Arabidopsis*, the miRNA inhibits mRNA translation (Chen, 2004; Zhang and Li, 2013). Interestingly, the miRNA target may activate the expression of its regulator miRNA, e.g. CUC2 and *MIR164a* (Nikovics et al., 2006). Therefore, regulation of genes by miRNA does not always imply a negative correlated expression between mature miRNA and the targeted transcripts. Plant *MIR* genes are often organized into multigene families in which the sequences of the precursors differ, but the mature sequences are almost identical, suggesting that they share some target mRNAs (Palatnik et al., 2007). Moreover, many *MIR* families are conserved between evolutionarily distant plant species, either targeting conserved genes or having different targets in different plant species (Jones-Rhoades, 2012). Small regulatory RNAs are major regulators of gene expression in plant development and in responses to various microorganisms such as beneficial mycorrhizal fungi (Bazin et al., 2013) and fungal (Park et al., 2014) or bacterial pathogens (Navarro et al., 2006). Plant miRNA may regulate the plant defense or the neoformation of specific structures during plant–microbe interactions (Combier et al., 2006; Park et al., 2014; Lee et al., 2015). Plant-parasitic nematodes induce the neoformation of feeding structures within host roots by inducing an extensive reprogramming of gene expression in the targeted root cells. The role of small noncoding RNAs in the plant–nematode interaction was established with the increased resistance to RKN and CN of *A. thaliana* mutants disrupted for miRNA or siRNA pathway (Hewezi et al., 2008; Medina et al., 2017; Ruiz-Ferrer et al., 2018). The development of sequencing technologies has made it possible to initiate studies of the role of plant miRNAs in this process in various plant species. This review provides an overview of current knowledge about of the conserved and species-specific plant miRNAs involved in responses to RKNs and CNs.

### Plant MicroRNAs Responding to RKNs

The identification of novel and differentially expressed (DE) miRNAs involved in plant response to nematodes is based principally on the sequencing of small RNAs (< 35 nt) from infected and uninfected root tissues. If three independent replicates *per* sample are available, the comparison can be performed directly, by digital expression profiling. Otherwise, sequencing identifies the miRNAs expressed in the samples analyzed, and the levels of these miRNAs are then compared between samples by reverse transcriptase–quantitative polymerase chain reaction (RT-qPCR). The miRNAs involved in the gall formation induced by RKN have been investigated in *Arabidopsis* dissected galls and uninfected roots, 3 (Cabrera et al., 2016), 7, and 14 dpi (Medina et al., 2017). This approach identified 62 miRNAs as DE in galls induced by *Meloidogyne javanica* at 3 dpi, and 24 miRNAs as DE in galls induced by *Meloidogyne incognita* at 7 and/or 14 dpi. Only two DE miRNAs with the same expression profile were common to these three stages of gall formation: miR390, which is upregulated in galls, and miR319, which is repressed in galls. Using RT-qPCR, identified 17 miRNAs as DE in tomato galls at one or more of the five developmental stages analyzed (Kaur et al., 2017), while Pan et al. (2019) identified 16 miRNAs as DE in whole cotton roots infected by *M. incognita* at 10 dpi (**Table 1**). A comparison of susceptible and resistant tomato cultivars identified five RKN-responsive miRNAs in the WT and/or the jasmonic acid–deficient *spr2* mutant at 3 dpi (Zhao et al., 2015). Some conserved miRNA families present similar expression profiles in galls from different plant species at similar time points. For example, the evolutionarily conserved miR159 is upregulated in *Arabidopsis*, tomato, and cotton galls at 10 to 14 dpi, and miR172 is upregulated in *A. thaliana* and tomato at 3 to 4 dpi (**Table 1**). The genes targeted by miRNAs have been identified by *in silico* prediction (Zhao et al., 2015; Cabrera et al., 2016; Pan et al., 2019) or by 5′ RNA ligase-mediated (RLM)–rapid amplification of cDNA ends (RACE) sequencing (Kaur et al., 2017). The expression profiles of genes predicted or known to be targeted by miRNAs were analyzed by transcriptomic analysis or RT-qPCR. A negative correlation between the levels of several DE miRNAs and their targeted transcripts, for miR156/SPB or miR159/MYB, for example, was observed in galls from *Arabidopsis*, tomato, and cotton (Zhao et al., 2015; Cabrera et al., 2016; Pan et al., 2019).

**Table 1:** List of functionally validated miRNAs differentially expressed in response to RKN and/or CN.

miRNA	Host plant	Infected material	Nematode species^a^	miRNA regulation^b^					References
				3 or 4	7	10	14	27-30	
miR159	*Arabidopsis*	Galls	*M. javanica*						Cabrera et al., 2016
		Galls	*M. incognita*						Medina et al., 2017
	Tomato	Roots							Zhao et al., 2015
		Roots							Kaur et al., 2017
		Roots	*G. rostochiensis*						Koter et al., 2018; Święcicka et al., 2017
	Cotton	Roots	*M. incognita*						Pan et al., 2019
miR172	*Arabidopsis*	Galls	*M. javanica*						Díaz-Manzano et al., 2018 (pre-miRNA)
		Galls	*M. javanica*						Cabrera et al., 2016 (mature)
		Roots	*H. schachtii*	*172c*	*172c*				Hewezi et al., 2008
		Roots	*H. schachtii*		*172a*				Hewezi et al., 2008
	Tomato	Galls	*M. javanica*						Díaz-Manzano et al., 2018
		Galls	*M. incognita*						Kaur et al., 2017
		Roots	*G. rostochiensis*						Koter et al., 2018
	Pea	Galls	*M. javanica*						Díaz-Manzano et al., 2018
miR319	*Arabidopsis*	Galls	*M. javanica*						Cabrera et al., 2016
		Galls	*M. incognita*						Medina et al., 2017
	Tomato	Roots	*M. incognita*						Zhao et al., 2015
		Roots	*G. rostochiensis*						Koter et al., 2018
	Cotton	Roots	*M. incognita*						Pan et al., 2019
miR390	*Arabidopsis*	Galls	*M. javanica*						Cabrera et al., 2016
		Galls	*M. incognita*						Cabrera et al., 2016
	Cotton	Roots	*M. incognita*						Pan et al., 2019
	Tomato and pea	Galls	*M. incognita*						Díaz-Manzano et al., 2018
miR396	*Arabidopsis*	Roots	*H. schachtii*	*396a*	*396a*				Hewezi et al., 2008; Hewezi et al., 2012
		Roots	*H. schachtii*	*396b*	*396b*				Hewezi et al., 2008; Hewezi et al., 2012
	Tomato	Roots	*M. incognita*						Zhao et al., 2015; Kaur et al., 2017
		Roots	*G. rostochiensis*						Święcicka et al., 2017
	Cotton	Roots	*M. incognita*						Pan et al., 2019
	Soybean		*H. glycines*						Noon et al., 2019
miR827	*Arabidopsis*	Roots	*H. schachtii*						Hewezi et al., 2016
	Cotton	Roots	*M. incognita*						Pan et al., 2019
miR858	*Arabidopsis*	Galls	*H. schachtii*						Piya et al., 2017

a nematodes species: RKN in yellow, CN in pink.

b expression pattern between 3 and 27-30 dpi; up-regulated in infected material in red; down-regulated in infected material in green.

Multiple miRNAs have been shown to be DE, but the functions of only four plant miRNAs in plant-RKN interactions have been validated to date. Functional validation involves the characterization of expression profile, often with reporter gene lines or by *in situ* hybridization, and analyses of the infection status of plants with modified expression or functions for either miRNAs (e.g. overexpression, KO or buffering “target mimicry” lines) or their targets (e.g. overexpression of a miRNA-resistant form, with a mutation in the miRNA target site or knockout lines). For example, miR319 is upregulated in tomato galls at 3 dpi, whereas its target, *TCP4* (*TEOSINTE BRANCHED 1∕CYCLOIDEA∕PROLIFERATING FACTOR 4*), is downregulated (Zhao et al., 2015). Tomato plants overexpressing a miR319-resistant *TCP4* have fewer galls and higher levels of endogenous JA, whereas the opposite effect is observed in lines overexpressing *Ath-MIR319*. These results suggest that the miR319/TCP4 module is essential in tomato galls by modulating the JA biosynthesis induced by RKN invasion (Zhao et al., 2015). miR159 is a conserved family of miRNAs upregulated in *Arabidopsis* galls at 14 dpi (Medina et al., 2017). Studies on transgenic GUS lines demonstrated the posttranscriptional regulation of MYB33, the main target of miR159, in *Arabidopsis* galls at 14 dpi. The *mir159abc* triple loss-of-function mutant displays enhanced resistance to RKN, with decreased numbers of galls and egg masses, demonstrating the role of the miR159 family in the response of *Arabidopsis* to *M. incognita*, probably through the regulation of MYB33. Furthermore, *in situ* hybridization has shown that *miR159* is also expressed in tomato giant cells (Medina et al., 2017) and a conserved upregulation of miR159 associated with a downregulation of MYB transcription factors has also been observed in galls from tomato (3 dpi and 13-15 dpi; Zhao et al., 2015; Kaur et al., 2017) and cotton (10 dpi; Pan et al., 2019). These results suggest that the function of the miR159/MYB module may be conserved in the galls *Arabidopsis*, tomato and cotton (Medina et al., 2017). The conserved auxin-responsive miR390 family is overexpressed in *A. thaliana* galls at 3, 7, and 14 dpi (Cabrera et al., 2016; Medina et al., 2017). In *Arabidopsis*, the cleavage of *TAS3* transcripts by miR390 generates secondary siRNAs (tasiRNAs) that induce post-transcriptional repression of the auxin-responsive transcription factors *ARF2*, *ARF3*, and *ARF4* (Marin et al., 2010). Cabrera et al. (2016) demonstrated the coexpression of *MIR390A* and *TAS3* in galls and giant cells at 3 dpi and the post-transcriptional regulation of *ARF3* by tasiRNAs in galls, in experiments comparing *ARF3* sensor lines sensitive or resistant to cleavage by tasiRNAs. Studies of *miR390a* and *tas3* loss-of-function mutants reported the production of fewer galls, suggesting that the miR390/TAS3/ARF3 regulatory module is required for correct gall formation (Cabrera et al., 2016). Finally, a role for the regulatory gene module composed by miR172 and the two transcription factors TOE1 (target of early activation tagged 1) and FT (flowering locus T) has been demonstrated in root galls during the formation of giant cells in *Arabidopsis* (Díaz-Manzano et al., 2018). The role for the miR172/TOE1/FT module has been first described during *Arabidopsis* flowering (Aukerman and Sakai, 2003). In *Arabidopsis* root, the 3′ strand of mature miR172 has been shown to be downregulated in galls at 3 dpi, whereas the pri-miR172 precursor is induced, and its target *TOE1* repressed, according to transcriptome data for microdissected *A. thaliana* giant cells at the same time point (Barcala et al., 2010). Consistent with the negative regulation of *FT* by TOE1, an induction of *FT* was observed in galls at 3 dpi. *Arabidopsis* plants expressing miR172-resistant *TOE1* or KO for *FT* were less susceptible to RKNs and had smaller galls and giant cells. Like miR390, miR172 is an auxin responsive microRNA. Auxin is a crucial signal for feeding site formation and parasitism. An enhanced auxin response has been observed in RKN feeding sites (Hutangura et al., 1999) and auxin has been identified in the secretion of RKNS (De Meutter et al., 2005). The function of miR390 and miR172 in the feeding site is probably a part of the auxin response.

### Plant Small Noncoding RNAs Responding to CNs

The identification and analysis of miRNAs involved in plant-CN interaction are based on the same approaches that the ones described above. Sequencing identified 30 mature DE miRNAs in *Arabidopsis* syncytia induced by *Heterodera schachtii* at 4 and 7 dpi, and qPCR analyses revealed inverse expression profiles for six miRNAs and their targets (Hewezi et al., 2008). A recent analysis of syncytia from tomato plants infected with *Globodera rostochiensis*, performed at 3, 7, and 10 dpi, identified between 200 and 300 miRNAs at each stage as DE (Koter et al., 2018). Reverse transcriptase–qPCR analyses revealed inversely correlated expression patterns for six miRNAs and their targets (Koter et al., 2018). Moreover, the expression of eight tomato miRNAs regulating defense-related proteins was specifically analyzed by qPCR at 3 and 7 dpi; an inverse correlation between the expression of these miRNAs and their targets in response to CN infection was observed (Święcicka et al., 2017). Finally, several studies have analyzed expression of soybean miRNAs in response to infection with *Heterodera glycines* by comparing expression levels in resistant and susceptible cultivars (Li et al., 2012; Xu et al., 2014; Tian et al., 2017). Tian et al. (2017) identified 60 miRNAs from 25 miRNA families as DE relative to uninfected roots in susceptible and/or resistant cultivars and validated the expression profiles of most of these miRNAs by qPCR. While most of the miRNAs identified by Tian et al. (2017) are upregulated in resistant lines relative to susceptible lines, the majority of miRNAs were downregulated in the study performed by Li et al. (2012). These discrepancies may reflect differences in resistance between these soybean cultivars or a technical bias related to the number of replicates analyzed in these two studies. A comparison of the expression profiles of conserved miRNAs in response to CN infection identified some miRNAs as DE, with the same expression profile, in several plant species. miR396b and the miR167 family were downregulated in *Arabidopsis* roots infected by *H. schachtii* at 4 and 7 dpi (Hewezi et al., 2008) and in tomato syncytia induced by *G. rostochiensis* at 3 and 7 dpi (Święcicka et al., 2017) (**Table 1**).

Three miRNAs DE in syncytia were validated by functional approaches. In *Arabidopsis*, miR396 was repressed at the onset of syncytium formation in roots infested with *H. schachtii* and upregulated at later stages, whereas its target transcription factors, the growth-regulating factors (GRF) *GRF1*, *GRF3*, and *GRF8*, displayed the opposite pattern (Hewezi et al., 2012). *Arabidopsis thaliana* mutants overexpressing miR396 have smaller syncytia and greater resistance to CN. These results suggest that the coordinated regulation of miR396 and *GRF1* and *GRF3* is required for correct syncytium development in *Arabidopsis*. Interestingly, a repression of the miR396 family associated with an upregulation of soybean *GRF* genes was observed in soybean syncytia induced by *H. glycines* at 8 dpi (Noon et al., 2019). A combination of 5′ RLM-RACE and a reporter gene approach demonstrated that the *GRF6* and *GRF9* genes were targeted by miR396 in syncytia. Transgenic soybean lines overexpressing pre-miR396 and GRF9 RNAi lines displayed similar decreases in the number of *H. glycines* females per root, reflecting an increase in resistance to CN. These results indicate that the miR396/GRF module is essential for *H. glycines* infection, and this role is conserved in *Arabidopsis* and soybean. Furthermore, the use of a reporter gene strategy made it possible to demonstrate an inverse correlation in the expression profiles of the conserved miR827 and its known target *NLA* (nitrogen limitation adaptation) during syncytium development in *Arabidopsis* (Hewezi et al., 2016). The overexpression of miR827 increased susceptibility to *H. schachtii*, whereas the expression of a miR827-resistant *NLA* decreased plant susceptibility. These results show that miR827 downregulates *Arabidopsis* immunity to *H. schachtii* by repressing *NLA* activity in the syncytium (Hewezi et al., 2016). Finally, a role for the miR858/MYB83 module has been established in *Arabidopsis* syncytia induced by *H. schachtii*, in which an inverse correlation of transcript levels was observed between miR858 and its target MYB83 at 7, 10, and 14 dpi (Piya et al., 2017). Modulation of the expression of these genes through gain- and loss-of-function approaches altered the *Arabidopsis* response to nematode infection, demonstrating a role for this module in syncytium formation.

### Conclusions and Perspectives

The results presented provide the first insigths into the function of miRNAs in the plant response to nematode infection. Except for miR390, expression profile of most miRNAs in feeding site shows heterogeneity (**Table 1**), with different expression profiles according to the type of feeding structures, the plant species, and/or the phase of development. Difference of expression in giant cell and syncytia may be explained by their distinct ontogenesis. Whether these variations of expression of plant miRNAs are directly induced by the nematode or are the results of modification of plant hormonal balance is a question that still needs to be investigated. The identification of the targets of these DE miRNAs and the biological pathways they regulate would improve our understanding of feeding cell development. Moreover, resistance genes of the nucleotide binding site-leucine-rich repeat (NBS-LRR) family genes are known to be targeted by miRNAs and phased siRNAs (reviewed by Fei et al., 2016). An inverse correlation on several tomato NB-LRR transcripts and their miRNA regulators has been evidenced after infection by CN (Święcicka et al., 2017). A better understanding of the role of miRNA in PPN feeding sites may lead to new methods of control for these organisms.

Most studies to date have focused on miRNAs, but few studies investigating the siRNAs expressed in roots infected with PPNs in *Arabidopsis* (Hewezi et al., 2017; Medina et al., 2018; Ruiz-Ferrer et al., 2018) have highlighted an overrepresentation in galls of 24 nt siRNAs known to be associated with RNA-directed DNA methylation. Two first studies of changes in DNA methylation have been performed in *A. thaliana* and soybean plants infected with CN (Rambani et al., 2015; Hewezi et al., 2017). These studies support a role for changes in DNA methylation in plant responses to PPN infection. Future combined studies of small RNAs, methylome and transcriptome should result in an integrative understanding of the epigenetic regulation of feeding site formation. Several intriguing questions remain unanswered: i) How do PPNs modify the expression of small RNA genes in the plant genome? ii) Do the small RNAs produced by nematodes play a role in the plant and vice versa? Genomes of several PPN species are now available (Cotton et al., 2014; Eves-van den Akker et al., 2016; Blanc-Mathieu et al., 2017; Masonbrink et al., 2019) and should be used to investigate the small RNAs produced by the nematode during parasitism. Finally, cross-kingdom RNAi (reviewed by Weiberg and Jin, 2015) probably also occurs during interactions between plants and PPNs. Integrative analyses of the small RNAs from both side of the interactions should shed light on this molecular dialog.

## Author Contributions

YN, BF, and SJ-P organized the study and wrote this manuscript. All authors have read and approved the manuscript.

## Funding

YN was supported by a Lebanese fellowship from Aazzée city. BF and SJ-P were supported by INRA and the French Government (National Research Agency, ANR) through the “Investments for the Future” LabEx SIGNALIFE: program reference #ANR-11-LABX-0028-01 and by the Plant-KBBE program NESTOR (ANR-13-KBBE-0003-06).

## Conflict of Interest

The authors declare that the research was conducted in the absence of any commercial or financial relationships that could be construed as a potential conflict of interest.
